# Dataset of 400,000 Prozorro public procurement tender records preserved in synchronization-feed order, with derived award-lot tables

**DOI:** 10.1016/j.dib.2026.113365

**Published:** 2026-10-19

**Authors:** Sushanta Paul

**Affiliations:** National Board of Revenue, Government of Bangladesh, Rajashwa Bhaban, Plot F-1/A, Agargaon, Sher-e-Bangla Nagar, Dhaka 1207, Bangladesh

**Keywords:** Electronic government, Open contracting, Ukraine, Temporal provenance, Sampling frames, Truncation sensitivity, Pseudonymization

## Abstract

Public procurement platforms often expose records through synchronization interfaces that order them by last modification rather than by the date of the procurement events they describe. This dataset preserves one such acquisition from Ukraine's Prozorro/OpenProcurement platform. On 13 July 2026, the public tender feed (API version 2.5) was walked from the modification-time offset 1 December 2023 until a ceiling of 400,000 tender identifiers was reached, and each tender was retrieved individually, with no logged failures. The retrieved tenders carry final modification timestamps between 1 and 26 December 2023. The deposit contains the identifiers in the exact order returned, a field-minimized JSON Lines snapshot of the 400,000 records retaining modification time and the buyer, lot, bid, award and award-complaint structure, and three derived tables: a tender-level feed-order table (400,000 rows), an eligible award-lot table (33,148 rows from 31,967 tenders and 8559 buyers) and a fixed November–December 2023 award-lot table (32,800 rows). Identifiers are replaced by consistent pseudonyms; names, contact details, monetary values, procedure types, item classifications, narrative text and documents are excluded. The repository also provides the acquisition script, offline derivation code, a separate re-derivation of the derived tables from the raw files, verification code with regression tests, pinned environments, checksums, a data dictionary and a provenance record listing known gaps. The data support methodological research on how modification-ordered interfaces and record ceilings shape retrieved collections, on stopping rules and cohort definitions, and on how derivation rules determine procedural markers such as single eligible bids and award-level complaint-type objects, as well as teaching reproducible handling of administrative records obtained through application programming interfaces.

Specifications Table

**Table:** 

Subject	Social Sciences
Specific subject area	Public procurement records, electronic government and the provenance of administrative data acquired through application programming interfaces.
Type of data	Table, Figure, Graph Compressed JSON Lines, CSV, plain text, JSON, Markdown, Python code Raw (field-minimized), Filtered, Processed, Analyzed
Data collection	A Python script run in Google Colab on 13 July 2026 walked the public Prozorro/OpenProcurement tender feed (limit=1000) sequentially from the modification-time offset 1 December 2023, following next-page offsets until 400,000 identifiers were collected, then retrieved every tender (60 concurrent threads, up to two attempts, 0 logged failures). Records were reduced to modification, buyer, lot, bid, award and complaint-type fields and pseudonymized. Derived tables and checks were produced offline with Python (pandas, NumPy, Matplotlib).
Data source location	Institution: State Enterprise “Prozorro”, operator of the Prozorro electronic public procurement system City/Town/Region: Kyiv Country: Ukraine Primary data source (secondary data): public Prozorro/OpenProcurement tender API, https://public.api.openprocurement.org/api/2.5/tenders (accessed 13 July 2026)
Data accessibility	Repository name: Zenodo Data identification number: 10.5281/zenodo.23348295 (version 1.3.0); all versions: 10.5281/zenodo.21374353 Direct URL to data: https://doi.org/10.5281/zenodo.23348295 Instructions for accessing these data: open access without registration; download the ZIP archive and see README.md. Derived tables, figures and results are regenerated and verified offline with python run_all.py, and the descriptive profile with python src/describe_dataset.py.
Related research article	None

## 1 Value of the Data

• The identifier file records the exact order in which a production procurement interface returned 400,000 tenders, and the tender feed-order table pairs each position with the record's final modification timestamp. Researchers can use them to examine how modification-ordered synchronization feeds and record ceilings determine which records a retrospective collection contains, without querying a live system whose contents change over time.
• Ten nested record prefixes (25,000 to 400,000 records), month-composition tables and the fixed November–December 2023 award-lot table provide fixed stopping points within one frozen collection for testing stopping rules, cohort definitions, denominators and truncation-sensitivity diagnostics.
• All derived tables, figures and results can be regenerated offline from the frozen inputs. Every distributed file can be checked against a SHA-256 manifest, a separate re-derivation rebuilds every field of the derived tables from the raw files and compares them row by row, and the provenance record states what the deposit cannot verify: the reduction of the full API responses to the minimized snapshot and the original feed order. The package can serve as a teaching and benchmarking example for documenting administrative data obtained through application programming interfaces.
• The minimized JSON Lines snapshot keeps the nested structure of tenders, lots, bids, awards and their complaint and claim objects, so users can apply their own eligibility rules, date definitions and lot definitions instead of the deposited derivations.
• The award-lot tables give, for 33,148 lots linked to tenders and buyers through consistent pseudonymous keys, the dates of the first award object and of the first active award, counts of eligible bids and of bid identifiers of any status, winner and non-winner counts, award-level complaint-type objects and award-to-modification intervals. These are procedural markers whose values depend on the stated derivation rules, and archive Tables S8, S9A and S9B show how alternative rules change them; they suit methodological work on derivation rules, sampling frames and truncation and are not validated indicators of competition, integrity or corruption risk. Without monetary values, procedure types, item classifications and narrative fields, they support only limited analysis of competition, corruption risk or procurement efficiency.

## 2 Background

Ukraine's Prozorro electronic procurement system [1] publishes its records through open application programming interfaces; its tender feed returns records sorted by modification time and pages through them with offsets meant for synchronizing a local copy of the central database [2]. The time at which a record was last changed is distinct from the time at which the events it describes took place [3], and administrative records gathered from operational systems carry no designed sampling frame [[4], [5], [6]]. A capped walk of such a feed selects records by their last modification, not by when procurement events occurred. The acquisition preserved here targeted awards made in January–June 2024 under a ceiling of 400,000 identifiers; the ceiling was reached among records last modified in December 2023, and the retrieved records contain no eligible award-lot in the targeted interval. The dataset keeps this acquisition as returned, so that feed order, modification time, award dates and record ceilings can be inspected directly. Procurement records also underpin competition and integrity indicators such as single bidding [7,8], subject to data quality [9] and standardization [10]; this dataset documents an acquisition and does not measure competition, integrity or corruption.

## 3 Data Description

The dataset is deposited in Zenodo as version 1.3.0 [11] and consists of one ZIP archive whose root directory, Reproducibility_Archive_Operational_Government_APIs, contains 65 files (Table 1). Fig. 1 maps the links between the principal data files. The frozen inputs are in data/raw: R23_ids_hashed.txt.gz lists 400,000 unique pseudonymized tender identifiers, one per line in feed order, so that line n corresponds to feed_rank n in the derived tables; R23_minimized.jsonl.gz holds one JSON object per identifier (Table 2); and R23_failures.txt is the retrieval-failure log, which is empty. The JSON Lines records are not stored in feed order and are joined to the identifier file on id. The derived tables in data/derived are described in Table 3; README.md lists the fields renamed since version 1.2.0, whose values are unchanged.

**Table 1:** Files in the repository archive (version 1.3.0). Paths are relative to the root directory Reproducibility_Archive_Operational_Government_APIs.

File	Content	Format
README.md	Dataset overview, coverage, directory map, licenses, reproduction steps and change log	Markdown
ARCHIVE_SCOPE.md	What the archive contains and does not contain; interpretation boundary	Markdown
PROVENANCE.md	Acquisition record, observed coverage, minimization, pseudonymization, known gaps and verification levels	Markdown
DATA_DICTIONARY.md	Definitions of every field in every data file	Markdown
DATA_RIGHTS_NOTICE.md	License matrix and scope with respect to the source records	Markdown
DATA_USE_NOTICE.md	Responsible-use guidance (not additional license conditions)	Markdown
LICENSE_DATA.txt	CC BY 4.0 license for data, tables, figures and documentation	Text
LICENSE_CODE.txt	MIT license for code	Text
MANIFEST.json	SHA-256 digest and size of the other 64 files	JSON
requirements.txt	Pinned pip dependencies of the reference environment	Text
environment.yml	Equivalent conda environment (Python 3.13)	YAML
run_all.py	One-command integrity check, re-derivation, regeneration, verification and tests	Python
run_all.sh	POSIX shell wrapper for run_all.py	Shell
acquisition/R23_POWERED.py	Original retrieval script, unmodified (not run by run_all.py)	Python
acquisition/README.md	What the retrieval script does and notes for users	Markdown
data/raw/R23_ids_hashed.txt.gz	Pseudonymized tender identifiers in exact feed order	gzip text; 400,000 lines
data/raw/R23_minimized.jsonl.gz	Field-minimized tender records (Table 2)	gzip JSON Lines; 400,000 objects
data/raw/R23_failures.txt	Retrieval-failure log	Text; empty
data/derived/tender_feed_order.csv	One row per tender in feed order (Table 3)	CSV; 400,000 × 6
data/derived/award_lots_with_feed_rank.csv	One row per eligible award-lot (Table 3)	CSV; 33,148 × 19
data/derived/fixed_cohort_award_lots.csv	Award-lots with first award-object month 2023-11 or 2023-12	CSV; 32,800 × 19
src/run_analysis.py	Derivation of data/derived and generation of outputs/tables, figures and results	Python
src/rederive_from_raw.py	Separate standard-library re-derivation of the derived tables from data/raw, compared field by field	Python
src/verify_outputs.py	Input-integrity checks and comparisons of outputs with fixed expected values	Python
src/verify_archive_manifest.py	Completeness and SHA-256 check against MANIFEST.json	Python
src/describe_dataset.py	Descriptive profile written to outputs/descriptive	Python
tests/test_analysis_outputs.py	13 regression tests	Python
outputs/tables/Table_2_nested_prefix_sensitivity.csv	Ten record prefixes: final dateModified, fixed-cohort lots, month shares, complaint-object proportion ratio	CSV; 10 rows
outputs/tables/Table_3_archive_conditional_complaint_contrast.csv	Lots with complaint-type objects among fixed-cohort lots with one and with two or more eligible bids	CSV; 2 rows
outputs/tables/Table_S1_analytical_field_dictionary.csv	Machine-readable award-lot field dictionary	CSV; 19 rows
outputs/tables/Table_S2_eligible_award_lots_by_award_month.csv	Eligible award-lots and complaint-type objects by first award-object month	CSV; 17 rows
outputs/tables/Table_S3A_fixed_cohort_counts_across_nested_prefixes.csv	Fixed-cohort lot counts at each prefix	CSV; 10 rows
outputs/tables/Table_S3B_fixed_cohort_complaint_contrast_across_nested_prefixes.csv	Complaint-object proportions for one versus two or more eligible bids at each prefix	CSV; 10 rows
outputs/tables/Table_S4_inherited_5pct_rule_cohort_by_prefix.csv	Months kept at each prefix by a rule requiring at least 5% as many lots as the most frequent month	CSV; 10 rows
outputs/tables/Table_S5_month_composition_by_prefix.csv	November and December lot counts and shares at each prefix	CSV; 20 rows
outputs/tables/Table_S6_award_to_modification_lag.csv	Award-to-modification interval summaries for fixed-cohort lots: all, by complaint-type object and by number of eligible bids	CSV; 5 rows
outputs/tables/Table_S7_reproducibility_archive_map.csv	Machine-readable archive map	CSV; 29 rows
outputs/tables/Table_S8_award_date_definition_sensitivity.csv	First award object versus first active award: lots without an active award and their award statuses, month assignment, November–December set and intervals	CSV; 46 rows
outputs/tables/Table_S9A_bid_status_rule_sensitivity.csv	Complaint-object contrast counting eligible bids or bid identifiers of any status	CSV; 4 rows
outputs/tables/Table_S9B_other_bid_statuses_on_one_eligible_bid_lots.csv	Statuses of the other bids on lots with one eligible bid	CSV; 8 rows
outputs/figures/Figure_1_three_clock_sampling_frame.png / .tif	Schematic of modification, award and complaint time in a capped feed walk	PNG; TIFF 600 dpi
outputs/figures/Figure_2_prefix_month_composition.png / .tif	Month composition of fixed-cohort lots across prefixes	PNG; TIFF 600 dpi
outputs/figures/Figure_3_prefix_estimate_trajectory.png / .tif	Complaint-object proportion ratio across prefixes	PNG; TIFF 600 dpi
outputs/figures/Figure_4_award_to_modification_lag.png / .tif	Award-to-modification intervals by presence of a complaint-type object	PNG; TIFF 600 dpi
outputs/results/claims.json	Headline counts and values used by the verifier and tests	JSON
outputs/results/analysis_summary.json	Structured summary of input audit, feed order, award-lots and prefixes	JSON
outputs/results/input_checksums.json	SHA-256 digests of the three raw inputs	JSON
outputs/results/ANALYSIS_MANIFEST.json	SHA-256 digests of inputs, code and generated outputs	JSON
outputs/results/run_log.txt	Log of the analysis run (reference environment)	Text
outputs/results/rederivation_report.txt	Report of the separate re-derivation	Text
outputs/results/verification_log.txt	Log of the input-integrity checks and expected-value comparisons	Text
outputs/results/pytest_log.txt	Log of the regression tests	Text
outputs/results/environment_log.txt	Interpreter and package versions of the reference-environment run	Text
outputs/descriptive/Figure_D1_temporal_coverage.png / .tif	Temporal coverage in two panels; data drawn as Fig. 2, Fig. 3 of this article	PNG; TIFF 600 dpi
outputs/descriptive/Figure_D2_file_structure_and_linkage.png / .tif	File structure and linkage; drawn as Fig. 1 of this article	PNG; TIFF 600 dpi
outputs/descriptive/Table_D1_field_summary.csv	Type, completeness, distinct values and range of every derived field	CSV; 44 rows
outputs/descriptive/Table_D2_records_by_modification_day.csv	Tender records per day of final dateModified	CSV; 26 rows
outputs/descriptive/Table_D3_award_lots_by_award_month.csv	Eligible award-lots per first award-object month	CSV; 17 rows
outputs/descriptive/descriptive_summary.json	Counts describing raw and derived files	JSON

**Fig. 1:**
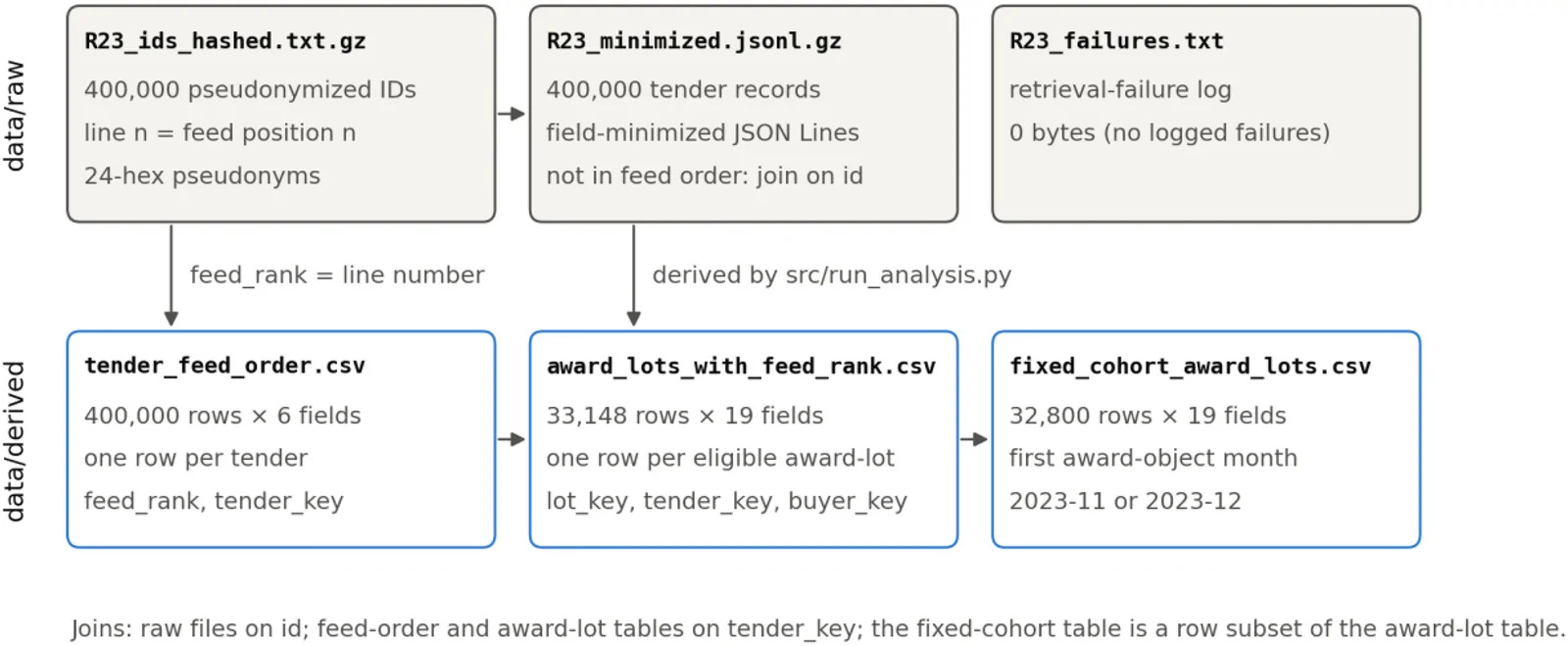
Structure and linkage of the principal data files. The identifier file fixes feed order; derived tables link through tender_key, and the fixed-cohort table is a row subset of the award-lot table. The diagram was drawn by plotting code written with Claude Opus 5.5 (Anthropic), which proposed the layout under the author's direction.

**Table 2:** Structure retained in data/raw/R23_minimized.jsonl.gz.

JSON path	Content
id	Tender pseudonym (24 hexadecimal characters)
dateModified	Tender modification timestamp held at retrieval (ISO 8601, +02:00)
procuringEntity.identifier.id	Buyer pseudonym
lots[].id	Lot pseudonyms; empty list for tenders without explicit lots
bids[].id, bids[].status	Bid pseudonym and status (active, deleted, invalid, unsuccessful)
bids[].lotValues[].relatedLot, .status	Lot pseudonym to which a bid value refers, and its status
awards[].status	Award status (active, pending, cancelled, unsuccessful)
awards[].lotID, awards[].bid_id	Lot and bid pseudonyms to which the award refers (may be null)
awards[].date	Award timestamp (ISO 8601 with Kyiv offset, +02:00 or +03:00)
awards[].complaints[].type	Object type: complaint or claim (status and submission dates not retained)

**Table 3:** Fields of the derived tables in data/derived.

Field	Table	Type	Definition
feed_rank	F, A	Integer	Position of the tender in the feed walk (1–400,000)
tender_key	F, A	Text	Tender key: first 20 hex characters of SHA-256 of tender:<id>
date_modified	F, A	ISO 8601	Final dateModified of the tender (+02:00 offset)
n_eligible_award_lots	F	Integer	Eligible award-lots derived from the tender
n_complaint_objects	F, A	Integer	Award-level objects of type complaint across the tender's eligible award-lots (F) or on the lot (A)
has_complaint_object	F	0/1	1 when n_complaint_objects (F) is greater than 0
lot_key	A	Text	Lot key from tender pseudonym and lot pseudonym (or __NOLOT__)
buyer_key	A	Text	Buyer key from the buyer pseudonym
first_award_object_date	A	Date	Earliest date among the lot's award objects of any status (calendar date as recorded)
first_award_object_month	A	YYYY-MM	Month of first_award_object_date; defines the fixed cohort
first_active_award_date	A	Date	Earliest date among the lot's active awards
first_active_award_month	A	YYYY-MM	Month of first_active_award_date
has_active_award	A	0/1	1 when at least one award of the lot is active
n_bids_lot	A	Integer	Eligible (active or pending) bids on the lot
n_bid_ids_any_status	A	Integer	Distinct bid identifiers linked to the lot, whatever their status
single_eligible_bid	A	0/1	1 when n_bids_lot equals 1
n_winners	A	Integer	Distinct bids on active awards for the lot (0 or 1)
n_non_winners	A	Integer	n_bids_lot minus n_winners, floored at 0
complaint_object_present	A	0/1	1 when at least one award of the lot carries an object of type complaint
award_object_to_modified_days	A	Decimal	date_modified (UTC) minus first_award_object_date at 00:00 UTC, in days
active_award_to_modified_days	A	Decimal	date_modified (UTC) minus first_active_award_date at 00:00 UTC, in days

F = tender_feed_order.csv (6 fields); A = award_lots_with_feed_rank.csv and fixed_cohort_award_lots.csv (19 fields each). first_active_award_date, first_active_award_month and active_award_to_modified_days are empty for the 2638 lots without an active award (2542 in the fixed cohort); all other fields are complete. Table_D1_field_summary.csv gives ranges.

All 400,000 records carry a final dateModified value between 1 December 2023 04:04:32 and 26 December 2023 15:44:20 (Kyiv time, UTC+02:00). In feed order, 99.663% of adjacent timestamp pairs are non-decreasing; the 1347 reversals are no longer than 1.817 s. Daily record counts range from 1247 (3 December) to 25,281 (20 December) (Fig. 2). The snapshot references 22,953 distinct buyer pseudonyms; 31,473 tenders have explicit lots, 32,106 have at least one bid and 388,204 have at least one award, and award objects carry 1300 objects in awards[].complaints (651 of type complaint and 649 of type claim). Applying the eligibility rule in subsection 4.3 yields 33,148 award-lots from 31,967 tenders and 8559 buyers, with first award-object dates from 7 December 2021 to 26 December 2023 falling in 17 distinct calendar months; November 2023 (10,261 lots) and December 2023 (22,539 lots) together account for 32,800 lots (Fig. 3). Of the 33,148 lots, 2638 have no active (successful) award at retrieval; 18,629 have exactly one eligible bid, 757 of which also carry deleted or invalid bids; and 521 carry at least one award-level complaint-type object (651 objects). The fixed November–December 2023 table covers 31,681 tenders and 8539 buyers, with 2542 lots without an active award, 18,521 lots with one eligible bid (751 of them also carrying deleted or invalid bids) and 444 lots carrying 516 complaint-type objects.

**Fig. 2:**
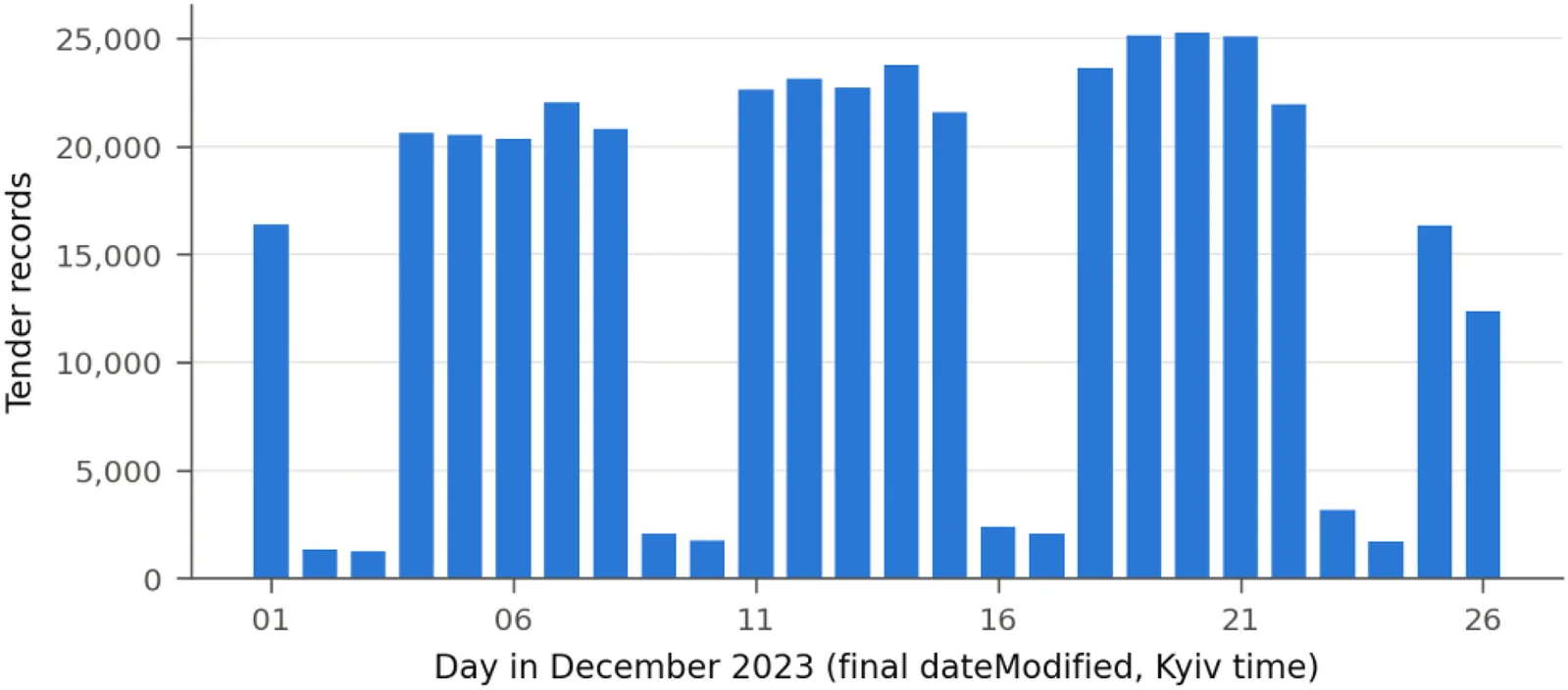
Tender records by calendar day of final dateModified (Kyiv time, UTC+02:00), 1–26 December 2023 (n = 400,000).

**Fig. 3:**
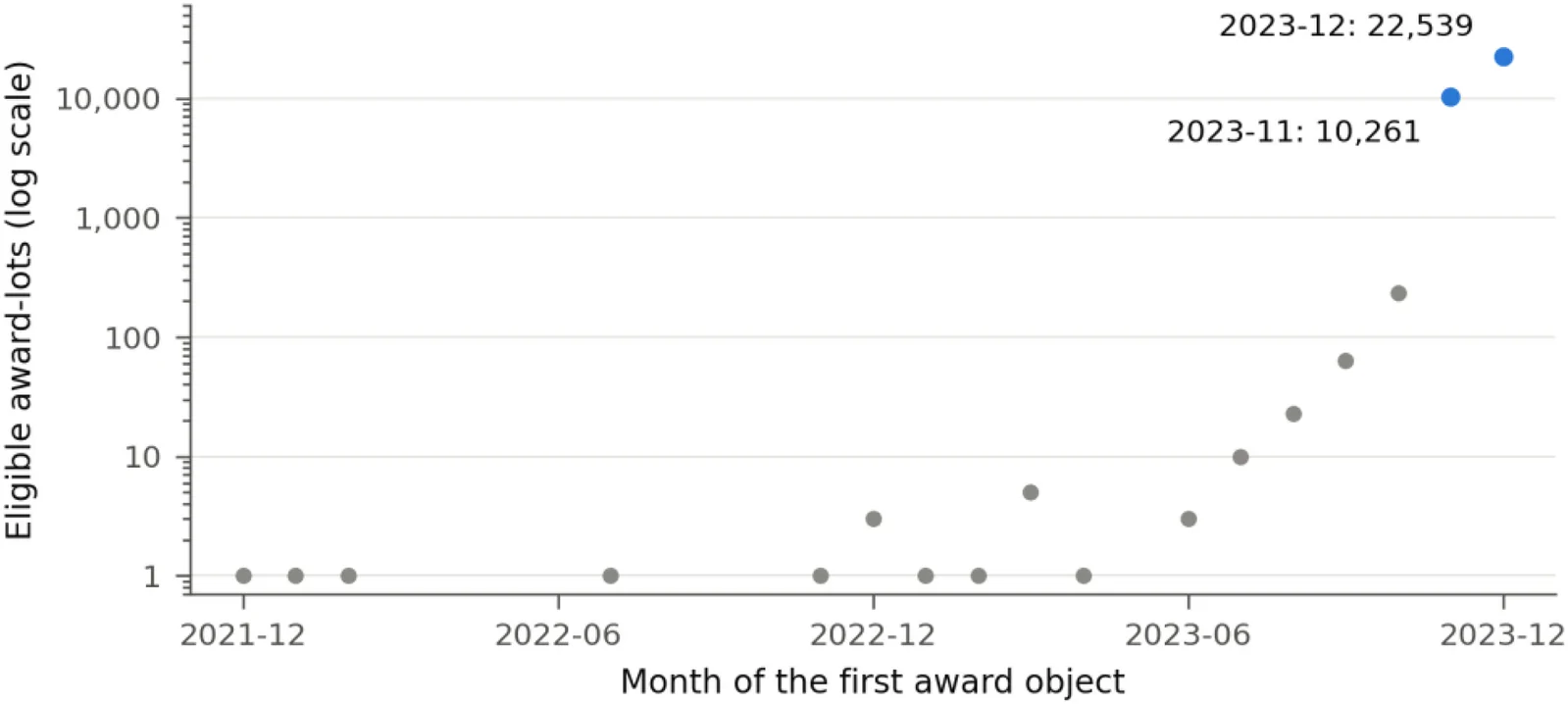
Eligible award-lots by month of the first award object on a logarithmic scale (n = 33,148). November and December 2023, which form the fixed cohort, are shown in blue; months without eligible award-lots are not plotted.

Folder outputs/tables contains the machine-readable tables generated by src/run_analysis.py, numbered independently of the tables and figures of this article. The nested-prefix table (Table_2_nested_prefix_sensitivity.csv) reports, for each of ten record prefixes, the final dateModified reached, the number of fixed-cohort award-lots, the shares of the final November and December lots already reached and the ratio of the proportion of lots with a complaint-type object among lots with one eligible bid to that among lots with two or more. Tables S1–S7 give the analytical field dictionary, award-lot counts by first award-object month, fixed-cohort counts and complaint-object contrasts by prefix, the months selected at each prefix by a rule keeping months with at least 5% as many lots as the most frequent month, month composition by prefix, award-to-modification interval summaries and the archive map. Table S8 compares the first award object with the first active award (it also gives the award statuses of the lots without an active award): 402 lots change month (335 from November to December 2023); assigning months by the first active award where one exists, and otherwise by the first award object, adds 56 lots to the November–December set and removes none (32,856 lots instead of 32,800), while counting only lots with an active award gives 30,314; for the 30,510 lots with an active award, the median award-to-modification interval is 7.57 days from the first award object and 7.53 days from the first active award (25th–75th percentiles 6.33–11.50 and 6.32–11.35 days). Table S9A repeats the fixed-cohort complaint-object contrast counting bid identifiers of any status: the proportions are 0.124% (22 of 17,770 lots with one bid) and 2.808% (422 of 15,030 with two or more), against 0.146% (27 of 18,521) and 2.920% (417 of 14,279) under the deposited rule (ratios 0.044 and 0.050); Table S9B lists the statuses of the other bids on lots with one eligible bid. Folder outputs/figures holds a schematic and three charts of these tables in PNG and 600-dpi TIFF format, and outputs/results holds the claims, summary, checksum and manifest files, the re-derivation report and the execution and environment logs listed in Table 1. Folder outputs/descriptive holds the descriptive profile produced by src/describe_dataset.py, including the data drawn in Fig. 1, Fig. 2, Fig. 3 and a completeness and range summary of every derived field (Table_D1_field_summary.csv). The folder acquisition contains the original retrieval script, and the root folder contains the documentation, license and environment files.

What can be verified from the deposit falls into three levels. File integrity is checked against MANIFEST.json. The derivation from the minimized snapshot is checked by regenerating the derived tables, by a separate re-derivation of every field from data/raw (outputs/results/rederivation_report.txt), by comparisons with fixed expected values and by regression tests; these checks test the implementation of the stated rules, not what the variables mean. The reduction of the full API responses to the minimized snapshot, and the original feed order, cannot be independently authenticated from the deposit, because the full responses and the pseudonymization program were not retained and no identifier mapping is deposited (subsection 4.2).

## 4 Experimental Design, Materials and Methods

### 4.1 Acquisition

The acquisition was performed with the Python script R23_POWERED.py (deposited unmodified in folder acquisition; SHA-256 88a6c414d66ac11616e8119c82ee734408ef1489c4b1c3c469b9b478e99ddab8) in a Google Colab notebook. According to the notebook's execution metadata, the run finished at 13:31 UTC on 13 July 2026 after 7381 s. The script worked in two stages. In the first, a single-threaded feed walk requested https://public.api.openprocurement.org/api/2.5/tenders?limit=1000&offset=2023-12-01 and then followed the offset returned in next_page, one page at a time, collecting identifiers until 400,000 had been gathered, a page's last dateModified passed 1 June 2025, a page came back empty or failed twice (75 s timeout), or no next offset was returned; the identifier ceiling was reached first, with the feed at 26 December 2023, so no page failed twice. In the second stage, the script requested every tender at /api/2.5/tenders/{id} using 60 concurrent threads and a 20 s timeout. A failed request (an HTTP error such as a rate-limit response, a timeout or another exception) was retried once after 0.5 s; identifiers whose second attempt also failed, or whose response contained no data, were written to R23_failures.txt as failures. The recorded console output reports 400,000 of 400,000 records fetched and no failures. Concurrency affected only the order in which records were written, not the feed order, which is kept in the identifier file. Requests used HTTPS with TLS certificate and hostname verification disabled in the script, so the server was not authenticated and interception during the run cannot be excluded retrospectively. As consistency checks across the two stages, every retrieved record carries a requested identifier, each identifier has exactly one record, and the dateModified values of the individually retrieved records follow the feed order obtained in the first stage (99.663% of adjacent pairs non-decreasing; reversals of at most 1.817 s); these checks do not authenticate the responses. The script declared an award-date target of 1 January to 30 June 2024 together with the 400,000-identifier ceiling. Because the feed is ordered by last modification, the ceiling was reached among records last modified in December 2023. Execution stopped in the first of the script's later analysis sections, which produce no deposited file, with KeyError: 'value' on an empty award-lot table, consistent with no award-lot falling in the target interval. The empty target interval is thus a property of the acquisition that the deposit preserves: under a record ceiling, a modification-ordered interface determines which award dates a collection can contain, whatever interval was intended.

### 4.2 Field minimization and pseudonymization

The full responses were first written to a checkpoint file and then reduced, in a later notebook step, to the fields listed in Table 2: tender identifier and dateModified; buyer identifier; lot identifiers; bid identifier, status and lot values (related lot and status); and award status, lot, bid, date and complaint type. All other fields, including names, contact details, supplier identities, monetary values, procedure types, item classifications, complaint statuses and dates, narrative text and documents, were removed. Tender, buyer, lot and bid identifiers were replaced by 24-character hexadecimal pseudonyms applied consistently across fields, so that every within-archive link is preserved. The full responses and the program that assigned the pseudonyms were not retained, so the mapping to live Prozorro identifiers cannot be reproduced from the deposit. The provenance record reports that a later offline comparison with the privately held field-reduced records found exact correspondence, under consistent identifier relabeling, for all 400,000 records and the 32,800 fixed-cohort lots; because those records are not deposited, this comparison cannot be repeated from the deposit. Derived keys are the first 20 hexadecimal characters of SHA-256 over the strings tender:<id>, lot:<id>|<lot> and buyer:<buyer id>, computed by src/run_analysis.py.

### 4.3 Award-lot derivation

Records are processed in feed order. A bid is eligible when its status is active or pending. The rule keeps bids that were still standing at retrieval and excludes deleted bids (proposals cancelled by the bidder [12], an operation that formerly set this status [13]), invalid bids (invalidated after a change to the tender conditions and not re-confirmed [14]) and unsuccessful bids; in this snapshot every counted bid has bid status active, and pending occurs only as a lot-value status. For tenders with explicit lots, an eligible bid counts toward each lot in its lotValues whose status is active or pending (a missing status is treated as active); for tenders without lots, eligible bids count toward a single tender-level unit. Awards without a lot identifier are ignored for tenders that have lots; in this snapshot every award in such a tender has one, so none is ignored. An award-lot is retained when it has at least one eligible bid and at least one award with a date. Awards of every status (active, pending, unsuccessful, cancelled) are award objects; only active awards are successful awards.

The field first_award_object_date is the earliest date among the award objects of the lot, whatever their status (the calendar date as recorded), and defines first_award_object_month; first_active_award_date is the earliest date among its active awards, and has_active_award marks lots with at least one active award. Of the 33,148 lots, 2638 (2542 of the 32,800 fixed-cohort lots) have no active award at retrieval: 1233 have only unsuccessful awards, 620 only pending awards and 785 a mix of two or more of the statuses unsuccessful, pending and cancelled (Table S8). In 2061 of the 30,510 lots with a dated active award, the first active award is later than the first award object; by construction it cannot be earlier.

The field single_eligible_bid marks lots with exactly one eligible bid at retrieval (n_bids_lot = 1). It does not show that only one bid was submitted or that only one bidder took part. The field n_bid_ids_any_status counts the distinct bid identifiers linked to the lot whatever their status; 757 of the 18,629 lots with one eligible bid also carry other bids: 750 only deleted bids, 3 only invalid bids and 4 both. None carries an unsuccessful bid.

The field n_winners counts distinct bids on active awards. Each of the 30,510 lots with an active award has exactly one active award, and its bid is one of the lot's eligible bids, so n_winners is 1 and n_non_winners, which equals n_bids_lot minus n_winners floored at zero, counts the lot's other eligible bids; the floor never applies. For lots without an active award, n_winners is 0 and n_non_winners equals n_bids_lot although the award process may not be complete, so winner-based analyses should be restricted to lots with has_active_award = 1.

The field complaint_object_present marks lots with at least one award-level object of type complaint, and n_complaint_objects counts these objects; objects of type claim are not counted. Because complaint status and submission dates were not retained, a complaint-type object does not establish that a complaint was formally submitted or accepted.

The field award_object_to_modified_days subtracts first_award_object_date, taken as 00:00 UTC, from the tender's dateModified converted to UTC, and active_award_to_modified_days does the same for first_active_award_date. Because the award date is a Kyiv calendar date taken as 00:00 UTC, an interval is negative, by up to 2 h (−0.083 days), when the tender was last modified within the first two hours of the award date in Kyiv time (UTC+02:00). This occurs for five lots in award_object_to_modified_days (minimum −0.081 days, about −1.9 h) and for one lot in active_award_to_modified_days (−0.077 days).

### 4.4 Fixed cohort and nested record prefixes

The fixed cohort keeps award-lots whose first_award_object_month is 2023-11 or 2023-12. The two months were chosen after inspecting the award months of the retrieved records, in which they hold 98.95% of eligible award-lots; the cohort was not pre-specified. It is a fixed reference set for the prefix diagnostics, conditional on tenders whose final modification fell between 1 December 2023 and 15:44:20 (Kyiv time) on 26 December 2023, when the ceiling was reached, and not a representative sample of procurement awarded in November–December 2023: tenders awarded in those months whose final modification fell before 1 December 2023 or after that time are absent. The feed sequence was cut at ten prefixes of 25,000, 50,000, 75,000, 100,000, 150,000, 200,000, 250,000, 300,000, 350,000 and 400,000 records; for each prefix the deposited tables record the final dateModified reached, retained fixed-cohort lots, month composition, counts of lots with a complaint-type object among lots with one and with two or more eligible bids, and the months that a rule keeping months with at least 5% as many lots as the most frequent month would select. The cohort was fixed for these comparisons after being selected from the full collection, and the prefixes are stopping points within one frozen collection: they show how a stopping point changes what a collection contains, not convergence toward a population value or performance across independent acquisitions. Table S8 recomputes month assignment, the November–December set and award-to-modification intervals with the first active award in place of the first award object, and Table S9A recomputes the fixed-cohort complaint-object contrast with bid identifiers of any status in place of eligible bids.

### 4.5 Offline regeneration and quality control

The reference environment is Python 3.13 with pandas 2.2.3, NumPy 2.3.5, Matplotlib 3.10.8, Pillow 12.3.0 and pytest 9.0.2 (requirements.txt, environment.yml). The command python run_all.py (i) verifies every distributed file against MANIFEST.json (64 files; the manifest excludes itself); (ii) rebuilds the three derived tables from data/raw with src/rederive_from_raw.py, a separate implementation that uses only the Python standard library and does not import src/run_analysis.py, and compares every field of every row with the deposited tables before anything is regenerated; (iii) regenerates the derived data, tables, figures and outputs/results/ANALYSIS_MANIFEST.json with src/run_analysis.py; (iv) runs src/verify_outputs.py, which checks raw-input integrity (fixed SHA-256 digests of the raw files, identifier and record uniqueness, one-to-one correspondence between identifiers and records, the empty failure log) and compares reported counts and ratios with fixed expected values; and (v) runs 13 regression tests, which guard against unintended changes but do not establish what the variables mean. No step uses the network. Step (i) checks file integrity and steps (ii)–(v) the derivation from the minimized snapshot; the reduction from the full responses and the original feed order cannot be checked from the deposit (Data Description). For version 1.3.0, python run_all.py and python src/describe_dataset.py were executed on a fresh copy of the deposit in the reference environment (Python 3.13.13 with the pinned packages): the re-derivation matched every field of every row, all checks and all 13 tests passed, and every regenerated file was byte-identical to the deposited one. The logs that run_all.py writes during this run (analysis, re-derivation, verification and test logs, and environment_log.txt with the interpreter and package versions) are deposited in outputs/results; elapsed times are masked so that the logs regenerate byte for byte. PNG and TIFF figure bytes can differ with other Matplotlib or Pillow versions. The descriptive profile (Tables D1–D3 and the figures in outputs/descriptive) is produced by src/describe_dataset.py; Fig. 1, Fig. 2, Fig. 3 of this article redraw Figure_D2 and the two panels of Figure_D1 from the same derived tables, as separate files with larger lettering for typesetting; the layout-only script used for this is not part of the deposit.

### 4.6 Use of generative AI in code and figures

All code in the deposit, the undeposited layout-only script that redrew Fig. 1, Fig. 2, Fig. 3 for typesetting and the version 1.2.0 documentation were written with the assistance of Claude (Anthropic) under the author's direction: the acquisition script and the derivation and verification code (src/run_analysis.py, src/verify_outputs.py, src/verify_archive_manifest.py and tests/test_analysis_outputs.py) with the versions of Claude available in July 2026, and the descriptive-profile script (src/describe_dataset.py), the layout-only script and the version 1.2.0 documentation with Claude Opus 5.5. For version 1.3.0, the changes to the derivation, verification and test code, the re-derivation script (src/rederive_from_raw.py) and the documentation were also written with Claude Opus 5.5. The re-derivation was written separately from src/run_analysis.py but with the same tool, so it checks the implementation of the stated rules, not the rules themselves. Fig. 2, Fig. 3 are data visualizations generated by this code directly from the deposited derived tables, and Fig. 1 is a schematic of the deposited files. All code was executed on the deposited data, and every data count reported in this article was also checked against a further re-derivation from data/raw that is not part of the deposit, carried out with Claude and reviewed by the author. No generative AI tool created, altered or imputed any data value.

## Limitations

The data come from one capped acquisition (400,000 records) of a feed ordered by modification time: tenders whose final modification, as held on 13 July 2026, fell between 1 and 26 December 2023, not a census or sample of tenders created or awarded in any period. The November–December 2023 cohort was selected after inspecting the retrieved records and does not represent procurement awarded in those months, and the nested prefixes are diagnostics within one collection, not evidence of convergence. Version histories and per-request HTTP logs were not collected, and the full responses and the pseudonymization program were not retained. TLS certificate and hostname verification were disabled during retrieval, so the server was not authenticated; users needing authenticated data should re-acquire with verification enabled. Without monetary values, procedure types, item classifications and narrative fields, procedures with different expected numbers of bids cannot be separated, and the data support only limited analysis of competition, corruption risk or procurement efficiency. The field single_eligible_bid depends on bid statuses at retrieval; complaint status and submission dates were not retained, so complaint-type objects need not all represent formally submitted complaints; and the 2638 lots without an active award had no successful award at retrieval.

## Ethics Statement

The author has read and follows the ethical requirements for publication in Data in Brief and confirms that the current work does not involve human subjects, animal experiments, or any data collected from social media platforms. The data derive from procurement records that the Prozorro system publishes openly; names and contact details were removed and identifiers pseudonymized before deposit. Pseudonymization removes direct identifiers from the deposit but does not prevent re-linkage to the public Prozorro records, which name buyers and suppliers: dateModified is retained to the microsecond and is distinct for almost every record (399,997 distinct values among 400,000 records), and dates, lot structures and buyer-level patterns provide further links. The snapshot contains no supplier identities, names or contact details, but some suppliers named in the public records may be natural persons, such as sole proprietors. The responsible-use guidance in DATA_USE_NOTICE.md asks users not to attempt to identify natural persons or to single out individual buyers or suppliers, and to treat single_eligible_bid and complaint_object_present as procedural markers, not as evidence of wrongdoing.

## Declaration of generative AI and AI-assisted technologies in the manuscript preparation process

During the preparation of this work, including its revision, the author used Claude (Claude Opus 5.5, Anthropic) in order to draft and revise the manuscript text, check the manuscript against the repository and the journal's guidelines, update the repository documentation and license files, and prepare the figures. The use of Claude in writing the data-acquisition and processing code is described in subsection 4.6. After using this tool/service, the author reviewed and edited the content as needed and takes full responsibility for the content of the published article.
